# Congenital Hairy Polyp of Posterior Tonsillar Pillar

**Published:** 2014-01-01

**Authors:** Bilal Mirza, Shahid Iqbal, Nabila Talat, Muhammad Saleem

**Affiliations:** Department of Pediatric Surgery, The Children's Hospital and the Institute of Child Health Lahore, Pakistan

**Keywords:** congenial hairy poly, posterior tonsillar pillar, neonate

## Abstract

Congenital hairy polyps are exceedingly rare congenital anomalies. We report a case of congenital hairy polyp arising from posterior tonsillar pillar which was excised with bipolar cautry.

## INTRODUCTION

Brown Kelly reported first case of congenital hairy polyp, a rare developmental anomaly with less than 150 cases reported so far. It has a predilection for female gender with an incidence of 1 in 40000 live births. It was previously mislabeled as teratoma, hamartomas, and dermoid. Majority of the cases present soon after birth with a pedunculated polypoid mass often extruded from mouth. Few cases in adults have also been reported. It has been associated with cleft palate, agenesis of the uvula, external auricle, facial hemihypertrophy, and left carotid artery atresia. The lesion often located in nasopharynx; other sites are palate, tongue, tonsils, ear, esophagus, and trachea [1-7]. We herein report a case of congenital hairy polyp arising from posterior tonsillar pillar.

## CASE REPORT

A 1-day-old female neonate, born at term to a primigravida, presented with a pedunculated skin covered finger like lesion extruding from within oral cavity. The lesion was associated with feeding difficulty however there was no respiratory distress present. General physical examination was unremarkable. Oral examination divulged a finger like projection appearing from posterior pharynx (Fig. 1). There was no cleft palate or other anomalies present. The patient was anesthetized by nasotracheal intubation as existence of intra-oral lesion constrained oral intubation. After employing mouth gag, the lesion was noted emerging from posterior tonsillar pillar. The lesion was excised with bipolar cautry. Bleeding was arrested with two stitches of absorbable suture. Patient had uneventful recovery and discharged on the next morning with full oral feeding established. Histopathology detected keratinizing stratified squamous epithelium lining the lesion with fibrous connective tissue, fatty tissue, and skeletal muscle fibers in deeper layers. Many sebaceous glands and hair follicles were embedded in the tissue.

**Figure:**
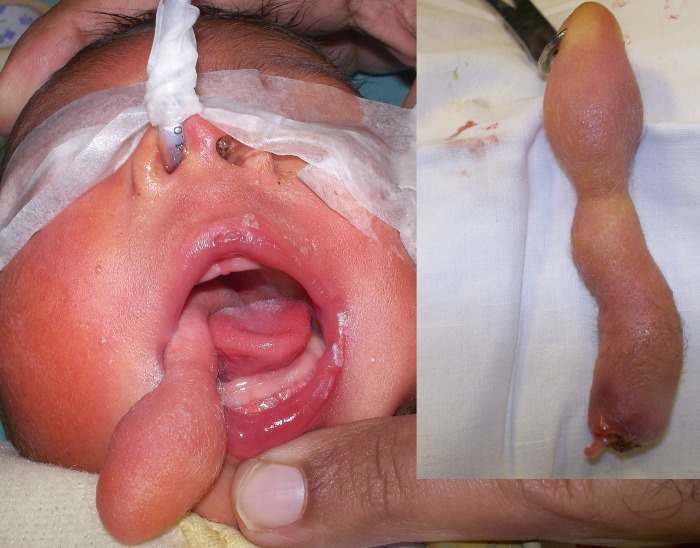
Figure 1: Figure showing congenital hairy polyp extruding from mouth. Inset shows excised lesion.

## DISCUSSION

The presentation of congenital hairy polyp is variable and characterized by respiratory difficulties owing to blockage of trachea by pedunculated mass which occasionally causes a ball valve like effect leading to cyanosis and acute airway emergency; feeding problems often occurs in case when it impinges the esophagus leading to drooling of saliva, vomiting, and inability to feed; nasal blockade; and a visible mass intra-oral or extruded from mouth. Few reports describe polyhydramnios; postnatal complications are failure to thrive, acute airway blockade, autoamputation of the hairy polyp, and hemorrhage [1,4]. MRI and CT scan are good diagnostic tools however final diagnosis is made on documentation of derivatives of ectoderm and mesoderm on histopathology [1,3]. Differential diagnoses are teratoma, dermoid, and epignathus. In our case, the child born without polyhydramnios and respiratory difficulties; however, there was difficulty with feeding the baby. At presentation, our preoperative diagnosis was oral teratoma; histology and literature search acquainted us of this entity.

Histopathologically, they are composed of skin with appendages (hair, glands) along with fibro-fatty tissue and skeletal muscles in deeper layers. No case of malignant transformation is reported to date. Treatment is surgical excision with cautry or suture ligation. Nasotracheal intubation should be preferred in case of intraoral lesion causing difficult oral intubation as we did in our case. Immediate postoperative concern is postoperative bleeding or airway problems [1-5]. A case of recurrent hairy polyp due to incomplete excision has been reported over a period of 6 years depicting a slow growing nature of this entity [7].

## Footnotes

**Source of Support:** Nil

**Conflict of Interest:** None

